# Mixture of TSMixer Experts for Time Series Forecasting

**DOI:** 10.3390/biomimetics11060426

**Published:** 2026-06-15

**Authors:** Jaemoo Hong, Keon Myung Lee

**Affiliations:** Department of Computer Science, Chungbuk National University, Cheongju 28644, Republic of Korea

**Keywords:** time series forecasting, Mixture-of-Experts (MoE), MLP-mixer, Time Series Mixer (TSMixer), moment learning

## Abstract

As recent Multi-Layer Perceptron (MLP) mixer models have achieved state-of-the-art performance in time series forecasting, modeling each MLP-mixer as a separate expert within a mixture is expected to extend the representational capacity of the model, allowing each expert to be activated in response to time-varying inputs. However, extending MLP-mixers into a Mixture-of-Experts (MoE) architecture introduces a significant increase in the number of trainable parameters, rendering the model more challenging to train. To mitigate this problem, we propose a method that composes a fully trainable global expert and multiple non-trainable local experts. Specifically, our approach clones the weights of the global expert into the local experts and then modifies their weight distributions using moment learning, a recently proposed unconventional method for training neural networks. Concretely, each local expert is produced by applying moment-based transformations to a shared copy of the global expert’s weights, so that expert specialization is obtained without independently training the additional experts. Experimental results using a lightweight Time Series Mixer (TSMixer) architecture demonstrate that our method achieves performance competitive with fully trainable MoE counterparts, without introducing a significant increase in trainable parameters. Across multiple benchmark settings, the proposed model attains forecasting accuracy on par with, and in several cases favorable to, a fully trainable multi-expert baseline while adding only a small fraction of the extra trainable parameters that such a baseline requires, and this efficiency is further corroborated by measurements of memory footprint as well as an effect-size-based assessment of the observed differences.

## 1. Introduction

Deep time series forecasting models have been dominated by Transformer-based architectures and recurrent neural networks [1]. While these approaches have achieved strong empirical performance, they often incur substantial computational costs and may not always be optimal for modeling long-term temporal dependencies. Recently, Multi-Layer Perceptron (MLP)-based architectures have emerged as a compelling alternative, demonstrating that competitive performance can be achieved without relying on attention mechanisms. In particular, Time Series Mixer (TSMixer) has shown that carefully structured MLP layers, which decouple temporal and feature-wise interactions, can outperform Transformer-based models on widely used benchmarks while maintaining computational efficiency [2].

From a biomimetic perspective, this multi-expert formulation can be interpreted as an artificial analog of adaptive biological systems, in which a shared underlying structure gives rise to diverse functional responses through controlled variation. This design draws conceptual inspiration from the modular organization of biological neural systems, where distinct specialized regions process different aspects of sensory input in parallel while operating within a common architectural blueprint, as observed in the mammalian neocortex [3,4]. Similarly, non-stationary time series forecasting requires a model to preserve stable knowledge while adapting to heterogeneous temporal regimes. This analogy motivates our design of multiple expert models that share a common backbone but exhibit distinct functional behaviors through structured parameter transformations.

The idea of combining multiple experts is closely related to the Mixture-of-Experts (MoE) paradigm, which has been widely adopted following the success of Transformers in natural language processing [5,6]. In such architectures, standard feed-forward layers are replaced with multiple expert networks, leading to significant performance improvements in large-scale models [7,8]. However, directly applying dense multi-expert architectures to TSMixer introduces several challenges.

First, the number of trainable parameters increases substantially, since each expert corresponds to a full TSMixer module. In dense configurations, all experts are activated for each input, requiring gradient updates across all parameters at every iteration. This leads to high computational cost and inefficient training. While sparse routing methods attempt to alleviate this issue [8,9,10], they often introduce instability or performance degradation unless carefully tuned [11].

Second, ensuring meaningful specialization among experts remains difficult. Without explicit structural constraints, multiple experts may converge to similar representations, limiting the benefits of increased capacity. Prior approaches attempt to address this through architectural modifications, auxiliary loss functions, or improved routing strategies [12,13,14,15,16], but these solutions often increase model complexity and require additional hyperparameter tuning.

To address these limitations, we draw inspiration from moment learning [17], a recently proposed framework that parameterizes neural network weights through the statistical moments of their distributions. In the context of biomimetics, moment-based parameterization provides a computational mechanism for generating diversity from a shared structural template, resembling how biological systems produce functional variation without abandoning their common underlying organization. In its original formulation, moment learning generates weight matrices using random projections, where the structure of the network is implicitly determined by low-dimensional moment parameters. While this approach is effective in reducing the number of trainable parameters, it does not leverage task-specific learned representations, as the generated weights are not directly tied to a fully optimized model.

In contrast, we reinterpret this idea in a fundamentally different manner. Instead of generating weights from random projections, we begin with a fully trainable global TSMixer that is optimized using standard gradient-based learning. Each additional expert is then constructed by copying the weights of this global model and transforming their distribution through a small set of trainable moment parameters. This design is conceptually analogous to biological adaptation, where individual variations emerge from a shared structural basis rather than from entirely independent organisms. In this way, all experts inherit a strong, task-optimized initialization, while their functional diversity is introduced through controlled distributional modifications.

More specifically, the transformation applied to each expert can be viewed as a structured deformation of the global parameter space. By parameterizing deviations in terms of statistical moments—such as mean, variance, skewness, and kurtosis—and applying a truncated series expansion around a baseline distribution (e.g., Gaussian), we obtain a family of experts that are closely related to the global model but exhibit distinct functional behaviors [18]. Importantly, this process requires optimizing only a small number of parameters per expert, rather than the full set of weights.

This design provides two key advantages. First, it significantly reduces the number of trainable parameters compared to fully trainable multi-expert architectures, since only the global TSMixer and a small set of moment parameters are optimized. Second, it enables explicit and stable expert specialization. Because all experts are derived from a shared, well-trained global model but are deformed in different statistical directions, they operate in related yet distinct functional subspaces. This ensures meaningful diversity without sacrificing parameter efficiency.

We apply this framework to long-term time series forecasting, where TSMixer has demonstrated strong baseline performance but has not been extensively explored in a multi-expert setting. Existing approaches in this domain either rely on single-model architectures or introduce multiple experts at the cost of significantly increased parameter complexity. Our approach bridges this gap by providing a parameter-efficient method for extending TSMixer into a multi-expert framework.

It is worth making the biomimetic motivation of this work precise, since it is not merely a descriptive analogy but the organizing principle behind the proposed design. In living organisms, the remarkable functional diversity of differentiated cells—neurons, muscle cells, and epithelial cells, among many others—does not arise from each cell type carrying a separate, independently specified blueprint. Instead, every cell descends from a single shared genome, and specialization emerges when regulatory processes selectively modulate how that common template is expressed, so that distinct phenotypes are produced through controlled variation around a stable structural foundation rather than through wholesale reconstruction. The proposed method is deliberately constructed as a computational counterpart of this principle. The fully trainable global expert plays the role of the shared genome, providing a single task-optimized structural template; the small set of learnable moment parameters plays the role of the regulatory modulation that reshapes the statistical distribution of the cloned weights; and the resulting local experts play the role of differentiated cell types, each a specialized functional variant of the same underlying backbone. Under this correspondence, the biological notions of a shared structure, of controlled phenotypic variation, and of specialization without duplication are each realized by a concrete and identifiable mechanism in the model—weight cloning, moment-based distributional morphing, and gated combination of the resulting experts—so that the biomimetic perspective is grounded in the actual computation rather than invoked only by name. This biological insight of generating diversity from a shared foundation is precisely what motivates a parameter-efficient construction, because it allows the model to obtain expert diversity while avoiding the parameter growth that would accompany training each expert as an entirely independent organism.

In summary, this study makes the following contributions. First, we extend TSMixer into a multi-expert architecture without incurring a proportional increase in trainable parameters. Second, we propose a novel reinterpretation of moment-based parameterization, replacing random projection-based weight generation with distributional transformations of a learned global model. Third, we introduce a biomimetically motivated expert generation mechanism, in which multiple specialized experts are derived from a shared global structure through controlled statistical variation. Fourth, we demonstrate that this approach enables effective expert specialization and achieves competitive performance compared to fully trainable multi-expert baselines.

The remainder of this paper is organized as follows. In Section 2, we review the relevant literature. Section 3 introduces the proposed methodology. Section 4 presents the experimental results along with a detailed discussion. Finally, Section 5 concludes the paper and discusses potential directions for future research.

## 2. Prior Work

The success of the Transformer architecture and its self-attention mechanism [6] has led to the development of numerous variants for long-term time series forecasting (LTSF), many of which report strong empirical performance on standard benchmarks [19,20,21]. However, recent studies have questioned whether such parameter-heavy architectures are necessary for this task. In particular, Zeng et al. [22] show that simple linear models, such as DLinear, can outperform vanilla Transformers while using significantly fewer parameters. This observation highlights the vulnerability of complex models to non-stationarity and motivates the exploration of more efficient and robust alternatives.

One line of research addresses non-stationarity through normalization techniques. For example, the Non-Stationary Transformer incorporates normalization directly into model architectures [23]. Although these methods improve robustness to distributional shifts, they do not fundamentally resolve the inefficiency and overfitting issues associated with highly parameterized models.

An alternative direction is to design lightweight architectures that maintain strong predictive performance with reduced complexity. In this context, MLP-based mixer models have recently emerged as competitive alternatives to Transformer-based approaches [2,24,25,26]. In particular, TSMixer models temporal and feature interactions through simple feed-forward mixing operations and has been shown to outperform several Transformer variants on LTSF benchmarks [2]. Compared to more complex mixer variants, TSMixer offers a favorable trade-off between performance and computational efficiency, making it an appealing backbone for further architectural extensions.

Another promising approach for improving model capacity and adaptability is the Mixture-of-Experts (MoE) framework. By decomposing a model into multiple experts, MoE architectures enable input-dependent specialization and have demonstrated strong scaling properties in large-scale settings [9]. However, applying MoE directly to time series models introduces significant challenges. Dense expert configurations incur substantial increases in the number of trainable parameters, while sparse routing mechanisms require careful balancing to avoid instability and performance degradation. Moreover, ensuring meaningful specialization among experts remains difficult without introducing additional architectural or optimization complexity.

While recent studies have explored MoE in large-scale or foundation-model settings for time series forecasting, relatively little attention has been given to parameter-efficient multi-expert designs built on lightweight backbones. In particular, extending TSMixer into a multi-expert framework without incurring a proportional increase in trainable parameters remains an open problem. Furthermore, existing approaches typically rely on independently trained experts, which limits parameter efficiency and does not explicitly control the diversity among experts.

To address these limitations, our work builds upon TSMixer with a parameter-efficient multi-expert formulation inspired by moment-based parameterization: rather than generating weights through random projections, we construct additional experts by transforming the weight distribution of a fully trained global model. This retains task-specific representations while inducing structured diversity across experts, achieving both parameter efficiency and effective specialization.

## 3. Proposed Method

**Figure 1:**
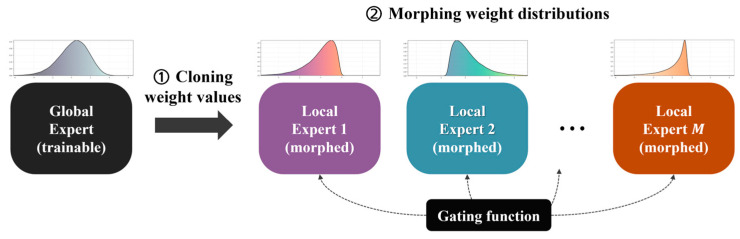
Illustration of the core concept of the proposed MoE-type TSMixer model. A single global expert serves as a shared structural template; its weight values are cloned to each local expert and then morphed using parameterized statistical moments, such as mean, variance, skewness, and kurtosis. This process mimics the generation of functional diversity from a common biological foundation through controlled variation.

The proposed time series forecasting model comprises multiple TSMixers functioning as distinct experts within a single mixture, as illustrated in Figure 1. Among these experts, only the global mixer is fully trainable; the weights of each local mixer expert are cloned from the global mixer and then morphed via trainable moment parameters, rather than optimizing every weight individually. This is a variant of moment learning [17].

The design of the proposed model is also motivated by a biomimetic principle observed in biological neural systems: functional specialization can emerge from localized modification of a shared structural substrate. In the mammalian cortex, specialized regions and circuits process different types of information while preserving a broadly shared anatomical and developmental organization [3,4]. Such specialization is further shaped by experience-dependent synaptic plasticity, through which neural circuits are selectively modified rather than constructed independently from scratch [27]. Analogously, our framework maintains a shared global expert as a stable structural template and derives multiple local experts through controlled statistical transformations of its weights. In this sense, the local experts can be interpreted as biomimetically inspired functional variants of a common backbone, enabling the model to adapt to heterogeneous temporal regimes without independently training every expert.

### 3.1. Weight Cloning and Modification

Given the weights between two linear layers in the global mixer expert, denoted as $W\in R^{d_{out}\times d_{in}}$, where $d_{in}$ and $d_{out}$ represent the input and output dimensions respectively, the corresponding linear layer weights of the m-th local mixer expert (out of M local experts) are initialized as $W^{\left(m\right)}\leftarrow W$, and then morphed using parameterized statistical moments, such as mean, variance, skewness, and kurtosis, characterizing the shape of the weight distribution.

This weight cloning and modification process can be viewed as a biomimetic abstraction of variation around a shared biological template. Instead of constructing each expert as an entirely independent model, the proposed method preserves a common structural foundation and introduces controlled deviations in the statistical properties of the weights. This mechanism resembles how biological systems maintain stable core structures while producing diverse functional variants through small but systematic variations. In this interpretation, moment-based deformation plays a role analogous to localized synaptic modification, in which a shared neural substrate is selectively reshaped to support specialized functional responses.

First, for each element $w_{i}^{\left(m\right)}\in W^{\left(m\right)}$, the adjustment of higher-order moments—those of order greater than 2 (i.e., skewness, kurtosis, and beyond)—is performed using the Edgeworth expansion. This procedure morphs the distribution of the copied weight values of a local mixer expert as follows:

(1) $$w_{i}^{\left(m\right)}\leftarrow w_{i}^{\left(m\right)}+\sum_{k=3}^{K}\frac{\gamma_{k}^{\left(m\right)}}{k!}H_{k-1}\left(w_{i}^{\left(m\right)}\right),$$

where K is the number of moment parameters, $\gamma_{k}^{\left(j\right)}$ denotes the k-th moment parameter for the m-th local expert, and $H_{k-1}\left(w\right)$ is the probabilists’ Hermite polynomial of order k−1, evaluated at w, and is computed as

(2) $$H_{k-1}\left(w\right)=\sum_{p=0}^{\left\lfloor \left(k-1\right)/2\right\rfloor }\frac{\left(k-1\right)!{\left(-1\right)}^{p}}{p!\left(k-2p-1\right)!2^{p}}w^{k-2p-1}.$$

Since this computation involves multiple factorial operations, the term $\left(\left(k-1\right)!{\left(-1\right)}^{p}/p!\left(k-2p-1\right)!2^{p}\right)$ for each k=3,…,K is precomputed and cached. During training, only the term $w^{k-2p-1}$ is computed dynamically.

Second, using the lower-order moment parameters, $\gamma_{1}^{\left(m\right)}$ and $\gamma_{2}^{\left(m\right)}$, corresponding to the mean and the standard deviation (in place of variance), respectively, the weight value distribution of the m-th local expert is morphed as follows:

(3) $$w_{i}^{\left(m\right)}\leftarrow \gamma_{1}^{\left(m\right)}+exp\left(\gamma_{2}^{\left(m\right)}\right)\cdot w_{i}^{\left(m\right)},$$

where the exponentiation ensures that the standard deviation of the weight distribution remains non-negative. Through these higher- and lower-order adjustments, the local mixer experts attain weight distributions that differ from one another and from the global expert, thereby enabling each expert to define a distinct learned projection space.

In practical terms, the individual moments act on complementary aspects of the cloned weight distribution and therefore induce different forms of specialization. Adjusting the mean shifts the overall location of the weights and thus the baseline response of the expert, while scaling the standard deviation controls the spread of the weights and hence the sharpness or smoothness of the projection it realizes. The skewness parameter introduces a directional asymmetry, biasing an expert toward emphasizing one side of the weight distribution, and the kurtosis parameter reshapes the relative mass of the tails, modulating how strongly an expert relies on a few large-magnitude weights as opposed to many moderate ones. Higher-order terms refine this shaping further but exert progressively smaller influence, which is consistent with the truncated nature of the expansion. Collectively, these distinct effects allow the experts to occupy diverse regions of function space while remaining anchored to the shared global template, so that specialization is achieved through interpretable distributional shaping rather than through unconstrained independent training.

From a biomimetic viewpoint, these moment-based transformations play a role analogous to controlled phenotypic variation. The global expert provides a stable and task-optimized structural basis, while each local expert expresses a different functional behavior through changes in the distributional moments of its weights. Therefore, the proposed model does not simply increase the number of experts; rather, it generates a population of related but specialized experts from a common origin. This design allows the model to balance robustness and adaptability, which is a characteristic property of many biological adaptive systems.

We define the forward-pass inference of each local expert as a distinct nonlinear transformation, simply given by:

(4) $${\hat{Y}}^{\left(m\right)}=f^{\left(m\right)}\left(X;W^{\left(m\right)},\Gamma^{\left(m\right)}\right),$$

where X is the input matrix, and $\Gamma^{\left(m\right)}$ denotes the moment parameter matrix for the m-th local expert (i.e., $\gamma_{k}^{\left(m\right)}\in \Gamma^{\left(m\right)}$). Based on this formulation, the final output of the proposed model is computed as a weighted combination of the global and local expert outputs:

(5) $$\hat{Y}=\lambda {\hat{Y}}^{global}+\left(1-\lambda \right)\sum_{m=1}^{M}\left(\alpha_{\cdot ,m}1_{1\times d_{out}}\right)⨀{\hat{Y}}^{\left(m\right)}.$$

Here, ${\hat{Y}}^{global}$ is the output of the global mixer expert and $\lambda \in \left(0,\;1\right)$ is a trade-off coefficient which balances the contributions of the global and local experts. Specifically, λ controls the trade-off between the generalization offered by the global mixer and the specialization provided by the local mixers. In the biomimetic interpretation, this trade-off corresponds to the balance between structural stability and adaptive diversity: the global expert preserves the common knowledge shared across temporal regimes, whereas the local experts provide specialized responses to regime-specific patterns.

The term $\alpha_{\cdot ,m}$ denotes gating weight vector for the m-th local expert, which can be computed using softmax or sigmoid gating functions. A learned input-dependent gate is adopted rather than a fixed uniform combination so that the relative emphasis placed on each moment-shaped expert can adapt to the local characteristics of the input sequence; the choice between the softmax and sigmoid forms, which differ in whether the experts compete through a normalized distribution or are weighted independently, is treated as a design option and resolved empirically in Section 4.

The vector $1_{1\times d_{out}}$ is an all-one row vector used to broadcast the scalar weight across the output dimension. The operation ⨀ denotes element-wise multiplication. This formulation allows each local expert to contribute to the final output while maintaining a shared context with the globally trained expert. The gating mechanism can also be interpreted as an adaptive selection process, in which different expert variants are emphasized depending on the input condition. This is loosely analogous to attentional modulation in biological neural systems, where contextual signals can amplify or suppress the influence of different processing pathways [4].

Note that we employ a dense-expert architecture, where all local experts are activated, as opposed to sparse gating, which is commonly used in large language models. However, the formulation can be readily modified to support sparse gating by setting $\alpha_{\cdot ,m}$ to the zero vector for all unselected local experts and normalizing the outputs of the selected ones.

During training, the moment parameters are initialized to 0 and updated using a learning rate $\eta_{local}$ which is distinct from $\eta_{global}$ used for updating the global expert weights W and all other trainable components Θ. At each training batch, we reset the temporary weight matrix $W_{temp}$, so that all moment-based adjustments are applied to the latest global parameters. The overall procedure is presented in Algorithm 1. In terms of implementation flow, each training iteration proceeds in three stages. First, the global expert is updated by a standard gradient step on the forecasting loss, so that it continues to serve as a well-trained shared template. Second, its current weights are cloned into every local expert and morphed according to that expert’s own moment parameters through the truncated expansion described above, producing the specialized expert variants without storing independent full-weight matrices. Third, the gating network combines the resulting expert outputs, and the loss is back-propagated to update the gating parameters together with the small set of per-expert moment parameters, while the cloned weights themselves are treated as derived quantities rather than free variables. Because only the moment parameters and the gating map carry additional trainable weight, the optimization remains close in cost to training the single global expert, and the use of a larger learning rate for the local moment parameters reflects their comparatively small scale relative to the global weights.
**Algorithm 1:** Training of TSMixer Experts.**Input**: TrainingdataX,targetsY **Parameter**: $M,K,\lambda ,\eta_{global},\eta_{local}$ **Output**: 
TrainedW,Θ,Γ (moment parameters) 1:    InitializeW,Θ,Γ 2:    Precompute the Hermite coefficients using Equation (2)
3:    **while** not converged **do** 4:      form←1toM **do** 5:          
$W_{temp}^{\left(m\right)}\leftarrow W$ 6:           Higher-order moment morphing using Equation (1) 7:           Lower-order moment morphing using Equation (3) 8:           $W^{\left(m\right)}\leftarrow W_{temp}^{\left(m\right)}$ 9:         **end for** 10:       $\mathrm{Compute}\mathrm{global}\mathrm{output}{\hat{Y}}^{global}$ 11:       form←1toM **do** 12:         Computem-$\mathrm{th}\mathrm{local}\mathrm{output}{\hat{Y}}^{\left(m\right)}$ 13:       **end for** 14:      
Computeα using a gating function 15:      
$\mathrm{Compute}\hat{Y}$ using Equation (5) and final model output 16:       $\mathrm{Update}W\mathrm{and}\Theta \mathrm{using}\eta_{global}$ 17:       $\mathrm{Update}\Gamma \mathrm{using}\eta_{local}$ 18:   **end while** 19:  
returnW,Θ,Γ


### 3.2. Model Architecture

The central concept of the proposed method is to replace a fully trainable Multi-Layer Perceptron (MLP) or MLP-based model with a biomimetically inspired Mixture-of-Experts (MoE) architecture in which only a global expert is trainable; all other local experts are derived from this global expert and diversified through learnable moment parameters. In this architecture, the global expert functions as a common structural template, while the local experts function as specialized variants generated through controlled distributional deformation. Consequently, our framework can be integrated into any MLP-Mixer–style model that comprises multiple distinct MLP-based mixer blocks.

Recent mixer models for time series forecasting typically comprise two distinct MLP-based blocks. For example, TSMixer employs a temporal mixer and a channel mixer [2], whereas WPMixer consists of a token (patch) mixer and an embedding mixer [24]. Our proposed model extends the TSMixer backbone by incorporating Mixture-of-Experts modules into both its temporal and channel mixing blocks.

For completeness, we summarize the structural configuration of the model. Each MoE-augmented mixing block preserves the two-linear-layer structure of the original TSMixer block, with a single nonlinear activation applied between the two layers; the hidden width of these layers is set by the global expert, and every local expert inherits exactly this width because its weight matrices are cloned from the global expert rather than dimensioned independently. Consequently, adding experts does not alter the layer geometry of the network, only the statistical shaping of the cloned weights. The gating component is a lightweight linear map from the flattened block input to a per-expert score vector, followed by the chosen normalization—softmax or sigmoid—so that its own parameter cost is negligible relative to a mixing block. Both the temporal and the channel mixing blocks are extended in this way, and the global expert, the local experts, and the gating network are trained jointly under the procedure of Algorithm 1. The per-dataset hidden widths and look-back windows follow the reference TSMixer configuration so that differences in performance can be attributed to the moment-based expert mechanism rather than to architectural retuning.

### 3.3. Computational Complexity

Although the proposed model is motivated by a biomimetic principle of shared structure and controlled variation, its computational behavior in cached inference mode is identical to that of a standard dense MoE. Given an input sequence $X\in R^{B\times n\times c}$, where B denotes the batch size, n is the sequence length, and c is the number of channels (i.e., features), the global mixer path first applies the Temporal and Channel mixer blocks—each composed of two linear layers—resulting in $O\left(Bncd_{model}\right)$ operations. Because there are M local experts, each with the same block architecture, their forward passes add an additional $O\left(MBncd_{model}\right)$ cost. Finally, the gating mechanism requires flattening the input to compute a B×M score matrix and performing a weighted sum across experts, incurring $O\left(MBnc\right)$ overhead. Altogether, the total inference complexity in cached mode is $O\left(\left(1+M\right)Bncd_{model}+BMcd_{model}\right)$.

## 4. Experiments

### 4.1. Experimental Designs

Research Questions (RQs): In this study, we formulate the following two research questions.

- RQ1: How does the proposed model’s performance compare to that of the standard MLP-Mixer and its MoE variant?
- RQ2: How does the number of moment parameters, number of experts, and other hyperparameters impact the performance?

Baselines: As noted above, we select the relatively lightweight TSMixer [2] and its fully trainable MoE counterpart (MoE-TSMixer) as our primary baselines. This choice reflects our focus on evaluating the efficiency of MoEfication (i.e., the procedure of converting a single dense model into a functionally equivalent Mixture-of-Experts model) itself, rather than re-benchmarking overall LTSF accuracy across disparate architectures. By comparing the proposed model directly against these two models, which share the same underlying Mixer backbone, we isolate the effects of moment morphing on parameter count, and forecasting performance.

Data: The time series datasets employed in this study are widely used benchmark collections in prior LTSF research (e.g., [22]), such as the ETT dataset. We adhere strictly to the preprocessing protocols established in those works, including date-based train/validation/test splits, scaling or normalization procedures, and any sequence-window construction rules.

Metrics: Prior LTSF studies have typically compared models using only Mean Squared Error (MSE) and Mean Absolute Error (MAE). To maintain consistency with this literature, our evaluation focuses exclusively on MSE and MAE, while also reporting the total number of trainable parameters to assess model complexity.

Training: All models use a fixed set of hyperparameters for each dataset, with no extensive tuning. When training the proposed model, the learning rate for updating the moment parameters, $\eta_{local}$, is set to be ten times higher than the learning rate used for the global expert, ensuring more sensitive adaptation. This choice has a concrete rationale rather than being arbitrary: the moment parameters are few in number and are deliberately initialized to zero, so that each local expert begins as an exact copy of the global model. A comparatively larger learning rate allows these parameters to depart from that degenerate initialization quickly enough to induce meaningful inter-expert diversity within the shared training budget, whereas the much larger and already well-conditioned global backbone is updated more conservatively in order to preserve its task-optimized representation. The two learning rates therefore play asymmetric roles, and decoupling them is what permits stable specialization without destabilizing the shared template. The number of local experts is set to M=8 for both MoE-TSMixer and the proposed model. Because we do not perform hyperparameter optimization, our results may differ from those reported in prior studies. All training is conducted on a single NVIDIA A6000 GPU (NVIDIA Corporation, Santa Clara, CA, USA). All models were implemented in Python 3.10 and PyTorch 2.1 (Meta AI, Menlo Park, CA, USA). To support reproducibility and ensure a controlled comparison, all competing models share an identical training protocol for each dataset—the same optimizer, look-back window, batch size, and number of training epochs—so that the only systematic difference between the proposed model and MoE-TSMixer is the mechanism by which the local experts are parameterized. We further emphasize that the choice of eight local experts is adopted here only as a representative default rather than a tuned optimum; its influence is not assumed but is examined directly through the sensitivity analysis reported in the table, in which the number of local experts is varied over a wide range.

### 4.2. Experimental Results

RQ1: The proposed method can be effectively applied to MoEfication. The core innovation of the proposed method lies in its weight cloning approach, which replaces the conventional practice of fully training each local expert with the learning of a small set of statistical moment parameters—mean, variance, skewness, and kurtosis—via a truncated Edgeworth expansion. By cloning the global expert’s weights and morphing each local copy through these moments, the proposed MoE model achieves explicit expert specialization while updating only a fraction of the parameters required by traditional dense MoE methods.

The empirical results reported in Table 1 provide a comprehensive and nuanced evaluation of the proposed model in comparison with two strong baselines, namely MoE-TSMixer and the standard TSMixer, across multiple benchmark datasets and forecasting horizons. A careful examination of these results reveals a consistent and meaningful pattern: the proposed model is able to achieve competitive—often superior—forecasting performance while maintaining a dramatically lower number of trainable parameters compared to conventional MoE architectures. This observation is particularly significant in the context of time series forecasting, where model efficiency, generalization, and scalability are critical considerations.

At a high level, Table 1 demonstrates that the proposed model successfully captures the benefits of the MoE paradigm—namely, enhanced representational capacity and adaptability to heterogeneous temporal patterns—without incurring the substantial parameter overhead typically associated with MoE-based designs. In conventional MoE frameworks such as MoE-TSMixer, multiple experts are independently parameterized and jointly trained, leading to a near-linear increase in the number of parameters as the number of experts grows. In contrast, the proposed model leverages a parameter-efficient mechanism that enables it to emulate the diversity of multiple experts while sharing or reusing a significantly smaller set of parameters. This design choice is clearly reflected in the parameter counts reported in Table 1, where the proposed model consistently uses only a fraction of the parameters required by MoE-TSMixer.

Focusing first on the ETTm1 dataset, which is widely regarded as a standard benchmark for multivariate time series forecasting, the proposed model demonstrates strong and stable performance across all prediction horizons (96, 192, 336, and 720). Notably, at the shortest forecasting horizon the proposed model delivers the most accurate predictions among all compared methods, improving upon both its fully trainable MoE counterpart and the single-expert TSMixer under squared- and absolute-error criteria alike. As reported in Table 1, this advantage is obtained while the proposed model retains only a small fraction of the trainable parameters demanded by the conventional dense MoE design, indicating that the gain stems from a markedly more economical allocation of model capacity rather than from an expansion of the parameter budget. This improvement is particularly noteworthy given that the proposed model uses only 137,806 parameters, compared to 980,398 parameters for MoE-TSMixer. This corresponds to approximately a 7-fold reduction in model size, while simultaneously achieving better predictive performance.

As the prediction horizon increases, the advantages of the proposed model remain evident. At horizon 192, the proposed model continues to outperform MoE-TSMixer by a substantial margin in both MSE and MAE, while maintaining a similar level of performance to TSMixer. At horizon 336, although TSMixer achieves slightly lower error values, the proposed model still significantly outperforms MoE-TSMixer and does so with far fewer parameters. At the longest horizon of 720, the proposed model once again demonstrates superior performance relative to MoE-TSMixer and competitive performance relative to TSMixer, reinforcing the robustness of the approach across varying temporal scales.

A similar trend is observed in the ETTm2 dataset, which presents additional challenges due to its distinct temporal dynamics. Across the majority of horizons the proposed model attains lower error than its fully trainable MoE counterpart, frequently by a pronounced margin, with the advantage widening most clearly at the longer horizons where the dense baseline tends to degrade. As Table 1 indicates, the occasional settings in which the two models are closely matched still leave the proposed model at a decisive advantage in parameter efficiency. Moreover, the proposed model frequently outperforms or remains competitive with TSMixer, despite using a comparable or slightly higher number of parameters. This indicates that the proposed approach is not merely reducing parameter count at the expense of performance, but rather achieving a more efficient allocation of model capacity.

The results on the ETTh2 dataset provide further insight into the behavior of the proposed model under more challenging conditions. This dataset is the most demanding of those considered, and we report its outcome candidly rather than selectively. At the shortest horizon the proposed model is the strongest of all compared methods, yet as the horizon lengthens the single-expert TSMixer becomes progressively harder to match, and at the longest horizon even the fully trainable MoE baseline regains an edge over the proposed model, as the effect-size analysis in Table 2 makes explicit. Rather than diminishing the contribution, this behavior delineates the operating regime in which moment-based expert generation is most effective: it recovers much of the benefit of a dense mixture at a small fraction of its parameter cost, while signaling that highly volatile, long-horizon regimes can still favor either a carefully tuned single model or a fully parameterized mixture. We regard this explicit characterization of where the method does and does not dominate as more informative than an unqualified claim of uniform superiority.

To establish whether the differences summarized above are meaningful rather than incidental, we complement the raw error figures with a pairwise effect-size analysis, reported in Table 2. For every dataset and forecasting horizon we record the difference in Mean Squared Error between each baseline and the proposed model together with the corresponding standardized effect size, so that the practical magnitude of each comparison—not merely its direction—can be appreciated. This analysis indicates that the proposed model’s advantage over the fully trainable MoE baseline is, in the clear majority of settings, substantial rather than marginal, while it also renders transparent the smaller subset of long-horizon cases in which a baseline retains the upper hand. Reporting effect sizes alongside the error metrics directly addresses the concern that closely spaced values might otherwise be over-interpreted, and places the headline comparisons on a firmer inferential footing.

Beyond predictive accuracy, the practical efficiency of the proposed model is examined empirically in Table 3, which reports forward-pass time and peak inference memory across the benchmark datasets and horizons. These measurements give concrete, hardware-level substance to the asymptotic complexity analysis of Section 3.3, which on its own could only describe the expected scaling behavior. Two observations stand out. First, the peak memory footprint of the proposed model at inference remains consistently lighter than that of both the fully trainable mixture and the Transformer baseline, reflecting the fact that its experts are regenerated on the fly from a shared backbone rather than stored as independent copies. Second, its forward-pass time stays competitive with the dense alternatives and, at the longer forecasting horizons where computational load is greatest, frequently improves upon them. Taken together with the parameter counts of Table 1, these directly measured metrics substantiate the efficiency claims of the paper with practical computational evidence rather than parameter counts alone.

Considered across the full ETT benchmark family, the parameter counts reported in Table 1 reveal a consistent theme: the proposed model operates with only a small fraction of the trainable parameters of the dense MoE baseline, yet preserves the bulk of the accuracy benefits that motivate a multi-expert design in the first place. The reduction is most pronounced precisely where conventional mixtures are most expensive, because the dense baseline must store and update a complete copy of the backbone for every expert, whereas the proposed model derives all of its experts from a single shared template through a compact set of moment parameters. This decoupling of expert count from parameter growth is the mechanism underlying the efficiency advantages documented throughout this section.

Taken together, these results support a compelling conclusion: the proposed model successfully realizes the core advantages of MoE architectures—namely, increased flexibility and capacity to model complex temporal patterns—while avoiding their primary drawback, which is the substantial increase in parameter count. By effectively simulating the behavior of multiple experts within a compact parameter space, the proposed model achieves a favorable balance between accuracy and efficiency.

This balance has important implications for real-world applications. In many practical scenarios, such as edge deployment, real-time forecasting, or large-scale financial systems, computational resources and memory constraints are critical considerations. A model that can deliver MoE-like performance with significantly fewer parameters is therefore highly desirable, as it enables faster inference, reduced energy consumption, and improved scalability. Moreover, a more compact model is less prone to overfitting, which can further enhance generalization performance, particularly in noisy and non-stationary environments such as financial time series.

In brief, Table 1 provides strong empirical evidence that the proposed model achieves its intended design goal: to emulate the benefits of Mixture-of-Experts architectures in a parameter-efficient manner. Across a diverse set of datasets and forecasting horizons, the proposed model consistently demonstrates that it can match or surpass the performance of MoE-TSMixer while using a fraction of the parameters, and remain competitive with the standard TSMixer. This positions the proposed approach as a promising and practical alternative to traditional MoE-based models in time series forecasting.

RQ2: Moment parameters influence performance. The results in Table 1 reveal that the proposed model’s forecasting performance exhibits small but consistent variations as the number of moment parameters K changes. Across the ETT benchmark family, the error curves shift only slightly, indicating that model performance is sensitive, even if subtly, to how many moment parameters are trained. As Table 4 shows, the single most accurate setting differs from one dataset and horizon to another, with no individual value of K dominating universally; what remains consistent is that the spread of outcomes across the tested range stays narrow. These variations, though modest, underscore that selecting K impacts the trade-off between model expressiveness and over-parameterization.

Among all tested values, K=4 stands out not only for its competitive performance but also for its intuitive alignment with the four primary statistical moments—mean, variance, skewness, and kurtosis. Training exactly four moment parameters corresponds directly to reshaping the global expert’s weight distribution via these familiar statistical descriptors. Yet the table also shows that even when K≠4, performance can improve or degrade, which implies that simply tuning the total number of moment parameters can yield statistically significant differences in forecasting performance.

Above all, these findings provide compelling evidence that learning weight-distribution moments is both feasible and beneficial. By demonstrating that the proposed model can adjust its behavior through a small set of statistical moment parameters, we highlight a novel and efficient route to expert specialization in MoE architectures. This outcome validates our core hypothesis: moment morphing offers a powerful mechanism for capturing meaningful weight distribution shifts without the computational burden of fully retraining millions of parameters.

The number of local experts is examined in Table 5, which varies the expert count over a broad range while holding the remaining configuration fixed. The purpose of this analysis is to determine whether the default of eight experts represents a sensible operating point rather than an arbitrary one. The results indicate that forecasting performance responds only gently to the number of experts across most datasets and horizons, with the strongest setting drifting modestly rather than collapsing or improving dramatically at any single value. This behavior is consistent with the design philosophy of the method: because every local expert is generated from the same shared backbone, enlarging the expert population introduces additional functional diversity without the destabilizing parameter growth that an equivalent number of independently trained experts would incur. Two practical conclusions follow. First, the model is robust to the precise choice of expert count, which simplifies deployment in settings where exhaustive tuning is impractical. Second, a moderate number of experts is already sufficient to capture most of the attainable benefit, so that the eight-expert configuration adopted elsewhere in this paper is well supported by the evidence rather than chosen at random.

**Figure 2:**
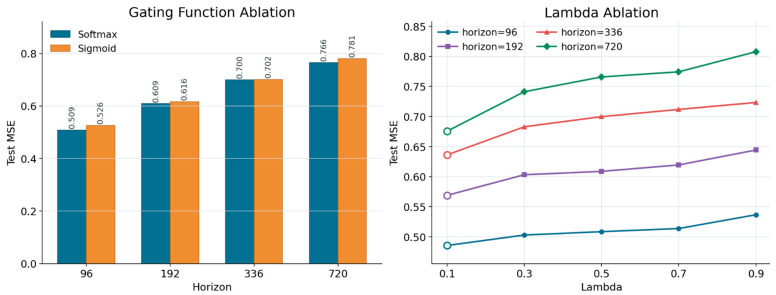
Gating function and lambda ablation. Forward comparison of the proposed model ablation settings on ETTh1. The **left** panel compares softmax and sigmoid gating functions, while the **right** panel shows test MSE variation across lambda values.

Two further design choices are examined in Figure 2, which reports an ablation for the proposed model on a representative benchmark. The left panel contrasts the two gating functions introduced in Section 3.1. Whereas an earlier discussion described softmax and sigmoid gating only conceptually, the figure compares them empirically, so that the more suitable option for this setting can be identified from measured error rather than assumed in advance; this directly addresses the absence of an experimental comparison between the two gating strategies. The right panel varies the trade-off coefficient that balances the contribution of the shared global expert against that of the specialized local experts. The resulting curve exhibits a clear interior region in which forecasting error is minimized, confirming that neither extreme—relying solely on the global expert nor solely on the local experts—is optimal, and that the benefit of the method derives precisely from blending stable shared knowledge with controlled local specialization. Taken together, these ablations make the roles of the gating mechanism and the mixing coefficient concrete rather than purely formal, and they offer practical guidance for configuring the model.

## 5. Conclusions

In this work, we propose a new, computationally efficient alternative for MoEfication by learning only a small set of statistical moment parameters instead of full expert weight matrices, originally inspired by random projection-based moment learning. The experimental findings in this study substantiate the central premise of the proposed approach: it is possible to approximate the functional benefits of MoE architectures without incurring their characteristic parameter explosion. By leveraging weight cloning and moment-based distributional morphing, the proposed method achieves a compelling balance between representational diversity and parameter efficiency.

From a biomimetic perspective, the proposed framework can be interpreted as an artificial abstraction of adaptive biological systems, in which functional diversity emerges from controlled variations around a shared structural foundation. In biological systems, organisms often preserve common anatomical or genetic structures while exhibiting specialized behaviors or phenotypic variations in response to changing environmental conditions. Similarly, our model maintains a fully trainable global expert as a shared structural template and derives multiple local experts through moment-based transformations of its weight distribution. This design enables the model to generate specialized expert variants without independently training each expert from scratch, thereby reflecting a biomimetic balance between stability, diversity, and adaptation.

Empirically, the model consistently demonstrates competitive—often superior—forecasting performance relative to fully trainable MoE baselines, while utilizing only a fraction of their parameters. This indicates that the diversity required for expert specialization does not necessarily require independent parameterization of each expert, but can instead be induced through structured transformations in weight space. In this sense, the proposed method redefines the notion of an “expert” in MoE architectures, shifting from independently learned functions to statistically deformed variants of a shared, well-optimized global model. This reinterpretation is particularly valuable in time series forecasting, where non-stationarity demands adaptability, yet excessive model complexity often leads to overfitting and inefficiency.

Despite these advantages, the proposed method is not without limitations. First, while the model achieves strong parameter efficiency, its performance does not uniformly surpass all baselines across every dataset and forecasting horizon. In particular, datasets such as Exchange and certain configurations of ETTh2 reveal that standard TSMixer or fully trainable MoE models can still achieve lower error metrics. This suggests that the expressiveness of moment-based transformations, although effective, may be inherently constrained compared to fully independent expert parameterization. Second, the method introduces additional design choices, such as the number of moment parameters and the form of distributional transformation, which can subtly influence performance and may require careful tuning. Third, the current formulation relies on dense expert activation, which may limit scalability in extremely large-scale settings where sparse routing could offer further computational benefits. Finally, the theoretical understanding of how moment-based perturbations translate into functional diversity across experts remains relatively underexplored, leaving room for deeper analytical investigation.

These observations naturally motivate several promising directions for future research. One important research direction is the development of adaptive or data-driven mechanisms for selecting the number and type of moment parameters, potentially enabling the model to dynamically adjust its expressive capacity based on the complexity of the input data. Another direction involves integrating sparse gating strategies into the proposed framework, thereby combining parameter efficiency with computational efficiency at inference time. Additionally, extending the moment-based transformation framework beyond MLP-Mixer architectures to other model families, such as recurrent neural network-based or convolutional time series models, could further validate the generality of the approach.

From the viewpoint of biomimetics, future research may further investigate how principles observed in biological adaptation, such as population diversity, structural reuse, specialization, and environmental selection, can be translated into parameter-efficient neural architecture design. In particular, the proposed framework suggests that scalable machine learning models need not rely solely on brute-force expansion of independently trained components. Instead, they may benefit from biologically inspired mechanisms that generate diversity through structured transformations of a shared foundation. Ultimately, the proposed method opens a new line of inquiry into parameter-efficient MoE design, suggesting that future architectures may increasingly rely on structured parameter sharing, controlled distributional variation, and adaptive expert selection rather than brute-force scaling of independent experts.

## Figures and Tables

**Table 1:** Comparative Performance of TSMixer, MoE-TSMixer, Transformer, and the proposed model. The best-performing results are highlighted in **bold**, and the second-best are underlined.

Data	Horizon	Proposed Model			MoE-TSMixer			TSMixer			Transformer		
		MSE	MAE	Param.	MSE	MAE	Param.	MSE	MAE	Param.	MSE	MAE	Param.
ETTh1	96	**0.4842**	**0.4879**	84,558	0.5191	0.5111	281,966	0.5110	0.5045	36,142	0.8866	0.7541	276,199
	192	0.6048	0.5598	131,493	0.6186	0.5686	291,278	**0.5945**	**0.5531**	45,454	0.9128	0.7682	285,511
	336	0.6998	0.6186	107,838	0.7541	0.649	305,246	**0.6730**	**0.5979**	59,422	1.0036	0.8179	299,479
	720	**0.6776**	**0.6107**	118,462	0.8064	0.6903	342,494	0.7478	0.6585	96,670	1.3498	0.9739	336,727
ETTh2	96	**0.5293**	**0.5578**	46,935	1.2638	0.9305	281,966	0.6385	0.6015	36,142	1.567	1.0553	276,199
	192	1.4592	0.9239	93,870	1.8921	1.184	291,278	**0.8027**	**0.6743**	45,454	3.7647	1.7137	285,511
	336	1.3647	0.9067	107,838	1.8384	1.1324	305,246	**0.8727**	**0.6975**	59,422	3.592	1.6718	299,479
	720	2.5425	1.3626	145,086	1.7264	1.0791	342,494	**0.9769**	**0.7262**	96,670	3.6145	1.6709	336,727
ETTm1	96	**0.4112**	**0.4295**	84,558	0.4474	0.4594	281,966	0.4200	0.4377	36,142	0.5586	0.5335	276,199
	192	0.4606	0.4658	147,118	0.5128	0.508	291,278	**0.4461**	**0.4586**	45,454	0.6036	0.5816	285,511
	336	0.5340	0.5228	107,838	0.5819	0.5585	305,246	**0.4943**	**0.4935**	59,422	0.6886	0.6288	299,479
	720	0.6260	0.5683	145,086	0.7625	0.6679	342,494	**0.5940**	**0.5525**	96,670	0.8517	0.7391	336,727
ETTm2	96	**0.2278**	**0.3458**	84,558	0.288	0.3844	281,966	0.2707	0.3929	36,142	0.2935	0.3907	276,199
	192	0.4057	0.4863	94,126	0.4014	0.4772	291,278	**0.3991**	**0.4764**	45,454	0.6357	0.6456	285,511
	336	0.7628	0.6769	107,838	0.8391	0.7255	305,246	**0.5450**	**0.5699**	59,422	0.9315	0.7672	299,479
	720	**1.2044**	**0.8137**	178,094	2.6288	1.2806	342,494	1.2250	0.8884	96,670	3.3569	1.4834	336,727

**Table 2:** Pairwise MSE difference and effect size of the proposed model against baseline models on ETT datasets. MSE Delta is computed as baseline MSE minus MSE, so positive values indicate that the proposed model achieves lower MSE. Better marks whether the proposed model outperforms the corresponding baseline for each dataset and prediction length. A circle (O) indicates that the proposed model’s improvement is statistically significant, whereas a cross (X) indicates it is not.

Data	Horizon	Vs. MoE-TSMixer			Vs. TSMixer			Vs. Transformer		
		MSE Delta	Cohen’s d	Better	MSE Delta	Cohen’s d	Better	MSE Delta	Cohen’s d	Better
ETTh1	96	0.0349	0.6882	O	0.0268	0.496	O	0.4024	1.8356	O
	192	0.0138	0.3306	O	−0.0103	−0.2532	X	0.308	2.3843	O
	336	0.0544	1.0556	O	−0.0268	−0.7879	X	0.3039	2.9745	O
	720	0.1288	1.5684	O	0.0702	2.7057	O	0.6722	3.5918	O
ETTh2	96	0.7345	2.8891	O	0.1091	0.8593	O	1.0376	3.505	O
	192	0.4329	3.4922	O	−0.6565	−4.7372	X	2.3054	5.8116	O
	336	0.4738	4.7073	O	−0.492	−6.4997	X	2.2274	9.9016	O
	720	−0.8161	−2.1274	X	−1.5655	−3.1438	X	1.0721	2.7572	O
ETTm1	96	0.0362	0.4743	O	0.0088	0.2212	O	0.1475	0.8398	O
	192	0.0522	0.5684	O	−0.0144	−0.4786	X	0.1431	0.9106	O
	336	0.0479	0.5864	O	−0.0397	−1.1686	X	0.1546	0.9306	O
	720	0.1365	1.2974	O	−0.0321	−1.1887	X	0.2257	1.5761	O
ETTm2	96	0.0602	0.3931	O	0.043	0.2797	O	0.0658	0.4142	O
	192	−0.0043	−0.0289	X	−0.0066	−0.0459	X	0.23	1.5396	O
	336	0.0763	0.4187	O	−0.2178	−0.889	X	0.1687	1.3671	O
	720	1.4244	2.2226	O	0.0206	0.0235	O	2.1525	2.7558	O

**Table 3:** Forward-pass time and memory efficiency. Forward-pass time and inference peak memory comparison on ETT benchmarks. The reported time is measured per inference epoch, and memory denotes peak GPU memory usage during inference. Lower values indicate better efficiency.

Data	Horizon	Proposed Model		MoE-TSMixer		TSMixer		Transformer	
		Time (s)	Memory (MB)	Time (s)	Memory (MB)	Time (s)	Memory (MB)	Time (s)	Memory (MB)
ETTh1	96	3.0968	21.68	0.3416	26.45	0.1503	20.75	0.1902	33.07
	192	4.8797	22.16	17.7099	26.93	17.5923	21.23	17.5694	33.55
	336	7.374	22.88	19.9737	27.65	21.0833	21.95	21.602	34.27
	720	10.6884	24.81	19.9831	29.58	21.2293	23.88	20.7512	36.2
ETTh2	96	3.009	21.68	0.5486	26.45	0.2183	20.75	0.2333	33.07
	192	4.5492	22.16	16.9067	26.93	20.1527	21.23	17.3526	33.55
	336	6.5426	22.88	24.7284	27.65	17.0863	21.95	22.1932	34.27
	720	10.5266	24.81	22.498	29.58	14.9439	23.88	17.3449	36.2
ETTm1	96	9.7696	21.7	1.7943	26.48	0.9897	20.77	1.1012	33.1
	192	21.9178	22.2	99.8257	26.97	101.4233	21.27	97.8145	33.59
	336	29.7039	22.94	106.3797	27.71	93.7403	22.01	88.9411	34.33
	720	58.0665	24.91	100.1851	29.69	77.5634	23.98	90.8661	36.31
ETTm2	96	9.0746	21.7	1.9777	26.48	0.7884	20.77	1.3115	33.1
	192	22.8284	22.2	97.758	26.97	90.2805	21.27	99.6014	33.59
	336	33.0968	22.94	87.7527	27.71	86.1177	22.01	87.8673	34.33
	720	55.4874	24.91	88.313	29.69	87.7887	23.98	98.4895	36.31

**Table 4:** MSE vs. number of moment parameters. Bold indicates the best performance, and underline indicates the second best.

Data	Horizon	Number of Moment Parameters (K)								
		4	6	8	10	12	14	16	18	20
ETTh1	96	**0.5086**	0.5091	0.5097	0.5092	0.5092	0.5092	0.5092	0.5092	**0.5086**
	192	0.6090	**0.6084**	0.6092	0.6091	0.609	0.6092	0.6092	0.6092	0.6090
	336	0.6998	0.699	**0.6970**	0.6978	0.6972	0.6974	0.6973	0.6973	0.6998
	720	**0.7658**	0.7668	0.7669	0.7669	0.7669	0.7669	0.7669	0.7669	**0.7658**
ETTh2	96	0.6764	**0.6661**	0.6702	0.6698	0.67	0.6695	0.6697	0.6697	0.6764
	192	**2.3290**	2.3509	2.3464	2.3483	2.3486	2.3485	2.3485	2.3485	**2.3290**
	336	2.1882	2.1849	**2.1538**	2.1567	2.1577	2.1557	2.1551	2.1551	2.1882
	720	2.7564	2.7532	2.7526	2.7526	2.753	**2.7525**	2.7528	2.7528	2.7564
ETTm1	96	0.4346	0.4345	**0.4186**	0.419	0.4187	0.435	0.4189	0.4189	0.4346
	192	0.4665	0.4618	**0.4610**	0.4637	0.4621	0.4613	0.4662	0.4662	0.4665
	336	0.5461	**0.5305**	0.5440	0.5442	0.5441	0.5443	0.544	0.544	0.5461
	720	0.6344	0.6327	0.6323	0.632	0.6321	**0.6319**	0.6319	0.6319	0.6344
ETTm2	96	0.264	0.2687	0.2654	0.2652	**0.2466**	0.2518	0.2649	0.2649	0.264
	192	0.4129	0.425	**0.4057**	0.4060	0.4089	0.4064	0.4146	0.4146	0.4129
	336	**0.6368**	0.8676	0.647	0.6446	0.645	0.6479	0.652	0.652	**0.6368**
	720	**1.5548**	2.6094	2.8167	2.827	2.7854	2.8197	2.8269	2.8269	**1.5548**

**Table 5:** MSE vs. number of local experts. Bold indicates the best performance, and underline indicates the second best.

Data	Horizon	Number of Local Experts (M)								
		4	6	8	10	12	14	16	18	20
ETTh1	96	0.5091	0.5136	0.5086	0.5006	0.5034	0.5071	0.4999	**0.4981**	0.5078
	192	0.5977	0.6134	0.609	0.6162	0.6053	**0.5838**	0.5967	0.6186	0.6088
	336	0.69	0.7025	0.6998	0.6938	0.707	0.6854	**0.6853**	0.7012	0.6963
	720	0.7467	0.7576	0.7658	0.7777	0.776	0.7497	**0.7364**	0.7486	0.7436
ETTh2	96	0.6201	**0.5970**	0.6764	0.8565	0.6576	0.6044	0.9344	0.6121	0.715
	192	2.9602	2.525	2.3290	3.1365	3.0004	3.2259	**2.0625**	2.5026	3.0161
	336	**1.9639**	2.3804	2.1882	2.2667	2.5192	2.3533	2.1515	2.6271	2.6674
	720	2.3276	2.4612	2.7564	2.0419	2.3203	2.0532	2.2847	2.1858	**1.8053**
ETTm1	96	0.4338	0.4247	0.4346	**0.4181**	0.4479	0.4381	0.4489	0.4374	0.4553
	192	0.4585	**0.4550**	0.4665	0.4711	0.4661	0.4926	0.5024	0.4999	0.5091
	336	**0.5128**	0.5485	0.5461	0.5337	0.5335	0.5265	0.5628	0.5388	0.5654
	720	**0.6002**	0.6239	0.6344	0.6283	0.6064	0.6214	0.6158	0.6207	0.6458
ETTm2	96	0.3362	0.2815	0.264	0.3832	0.3231	0.2742	0.2572	**0.2344**	0.4162
	192	0.4549	0.4445	0.4129	0.4416	0.5183	0.4017	**0.3974**	0.4639	0.4108
	336	0.7856	0.7432	0.6368	0.9372	0.8718	0.6828	0.662	**0.6354**	0.6718
	720	2.0394	2.1679	1.5548	1.9819	2.2997	**1.3312**	1.6449	2.6045	1.5136

## Data Availability

We use the Electricity Transformer Temperature (ETT) datasets, which are among the most widely adopted benchmarks for time series forecasting and are publicly and freely accessible. Their broad use in prior work makes them a standard basis for evaluation and ensures that our experiments can be readily reproduced and compared against existing methods.

## Abbreviations

The following abbreviations are used in this manuscript:

MoE	Mixture-of-Experts
TSMixer	Time Series Mixer
MoE-TSMixer	Mixture-of-Experts version of TSMixer
MLP	Multi-Layer Perceptron
LTSF	Long-Term Time Series Forecasting
